# Nurses’ experiences of the attributes of the organizational citizenship behavior: a qualitative content analysis

**DOI:** 10.1186/s12913-024-10939-8

**Published:** 2024-04-26

**Authors:** Rahimeh Khajoei, Mozhgan Jokar, Parvaneh Vasli

**Affiliations:** 1 School of Nursing and Midwifery, Shahid Beheshti University of Medical Sciences, Tehran, Iran; 2 Department of Community Health Nursing, School of Nursing and Midwifery, Shahid Beheshti University of Medical Sciences, Vali-Asr Ave., Ayatollah Hashemi Rafsanjani Cross Road, Tehran, Iran

**Keywords:** Nurse, Human resources, Qualitative study

## Abstract

**Background and aim:**

Organizational citizenship behavior (OCB) among nurses, as the key human resources in healthcare systems, is of paramount importance to nursing care quality and patient outcomes. The present study was to reflect on Iranian nurses’ experiences of OCB.

**Methods:**

This qualitative study was completed in Iran from December 2022 to October 2023. In total, 20 nurses involved in hospitals, meeting the inclusion criteria, were recruited by purposive sampling with maximum variation. The data were then collected using 20 semi-structured interviews, each one lasting 30–60 min, and finally analyzed through qualitative content analysis.

**Results:**

The data analysis revealed the nurses’ experiences of OCB under nine subcategories and three main categories, including (i) “helping behavior”, comprised of four subcategories of helping colleagues at work, helping colleagues outside of work, boosting morale, and creating a culture of support and appreciation, (ii) “extra-role behavior” with two subcategories of cooperation in advancing tasks, and creativity and efforts to promote services, and (iii) “contribution to professional growth and development”, consisting of two subcategories of individual professional development and support for colleagues’ professional development.

**Conclusion:**

Nursing managers and instructors can use the study results to enhance nurses’ OCB by evaluating and employing nurses, and incorporating OCB into nursing curricula and continuous training programs.

**Supplementary Information:**

The online version contains supplementary material available at 10.1186/s12913-024-10939-8.

## Introduction

The nursing industry at this time is acting in response to the emerging challenges triggered by population aging, chronic disease prevention and management, and birth control policies. It also aims to accelerate the delivery of high-quality services to cover the whole life cycle and transform many conditions from practicing treatments to meeting physical and mental health needs in patients [1]. In this line, nurses’ extra-role behavior, typically known as organizational citizenship behavior (OCB), seems to be vital for healthcare organizations [2]. OCB refers to employees’ cooperative behavior beyond their prescribed duties. This behavior is performed without the expectation of rewards or benefits but to support organizational effectiveness and success [3].

Given that OCB, as a factor affecting behaviors, is able to influence employees’ attitudes and interactions in order to provide high-quality services, its improvement makes it possible to augment the effectiveness of hospitals as healthcare organizations and increase patient satisfaction. This type of voluntary behavior is also among the significant factors that can help promote organizational culture, e.g., patient safety in clinical settings [4]. As well, OCB improves employees’ efficiency and participation, encourages teamwork and inter-organizational cooperation, minimizes costs of mistakes, and generally provides an appropriate work environment [5].

## OCB: Definitions and dimensions

The term OCB was initially suggested by Dennis Organ in the early 1980s [6] as a variable for increasing organizational effectiveness [7]. He further defined this concept as “individual behavior that is discretionary, not directly or explicitly recognized by the formal reward system, and that in the aggregate promotes the effective functioning of the organization” [8]. He shortly revised this definition and illustrated it as work-related behavior beyond normal tasks that provide support for social or psychological situations surrounding individuals [9]. OCB as a set of behaviors that do not develop in formal job descriptions, but facilitate the functioning of organizational tasks. Based on this background, OCB is not defined by official rules in an organization and is not even associated with formal rewards [10].

Considering its dimensions and different types, OCB has been investigated by numerous researchers [11]. As a pioneer in developing OCB, Organ [8] recapitulated it into five dimensions, including altruism, courtesy, conscientiousness, sportsmanship, and civic virtue. In this sense, altruism represented advantageous acts that could significantly contribute to the performance of other employees and smooth the progress of good working relationships. Courtesy included behaviors that could help colleagues and prevent problems. As well, conscientiousness signified a set of behaviors that could rise above the minimum requirements of organizational roles. Sportsmanship also meant employees’ tolerance of substandard organizational conditions with no complaints and attempts to get rid of problems unrealistically. Besides, civic virtue was characterized by behaviors indicating employees’ concerns and interests regarding organizational life [12]. In a review study, Podsakoff et al. [10] sought to identify 30 types of OCB with conceptual overlaps in some cases. He further placed them under seven themes, viz., (i) helping behavior, (ii) sportsmanship, (iii) organizational loyalty, (iv) organizational compliance, (v) individual initiative, (vi) civic virtue, and (vii) self-development. Likewise, Vigoda [13] divided OCB into two types, namely, (i) avoidance that might harm an organization and colleagues, and (ii) helping others actively. McNeely and Meglino [14] also categorized OCB into two groups, those focused on helping colleagues and customers, and the one leveled at helping an organization. In this line, Williams and Anderson [15] further suggested that OCB could fall into two categories, i.e., (i) OCB-individual, and (ii) OCB-organizational. The first category consisted of behaviors demonstrated in the workplace aimed at other people, while the second category included a set of behaviors directed toward an organization in general.

## Literature review

In recent years, numerous studies have been conducted on nurses’ OCB, primarily using descriptive and cross-sectional research designs. Dargahi et al. [16] investigated OCB in Iranian nurses and found that most of the nurses exhibited OCB behaviors. Similarly, Taghinezhad et al. [17] conducted a study in Iran and found that organizational commitment was the strongest predictor of nurses’ OCB, followed by perceived procedural justice. Another study indicated that organizational citizenship behavior (OCB) had a direct and indirect impact on family-centered care, with the mediating role of multiple commitments, including commitment to the leader and commitment to the organization [18]. In a separate study conducted in Italy, the results showed that professional competency is positively associated with both job satisfaction and organizational citizenship behavior [19]. Kazemipour et al.’ study [20] in Iran demonstrated that workplace spirituality predicts nurses’ OCB and affective organizational commitment. Additionally, a study in China showed that work engagement and role conflict can affect OCB [21].

Although there have been abundant studies on OCB among nurses, this concept has not yet been explored in a qualitative manner. In addition, this type of voluntary behavior is likely to be influenced by languages, norms, thoughts, and values in different communities, but there is little information about this phenomenon at the global and cross-cultural levels [22]. Moreover, there are some controversies that OCB cannot be measured quantitatively, and it should be investigated with qualitative methods, such as observation and interview [23]. As qualitative research methods seek to discover and understand the inner world of individuals, and the truth about each person is typically based on experiences, researchers could thus reflect on experiences and grasp the meaning of many phenomena, including OCB, from others’ perspectives [24]. In light of this, the present study aimed to explain the Iranian nurses’ experiences of OCB.

## Methods

### Study design

This qualitative study was conducted on nurses’ experiences of OCB in Iran from December 2022 to October 2023, using qualitative content analysis to analyze the data and interpret their meanings. Thus, the qualitative content analysis recruited here could represent a systematic and objective way to describe and measure OCB [25].

### Setting

The study was conducted in the hospitals affiliated with Shahid Bahshti University of Medical Sciences (SBMU), one of the three medical universities in Tehran, Iran, which has 12 general teaching hospitals. Like other regions in Iran, nursing staff-related challenges are faced by hospitals affiliated with SBMU. The reasons for the shortage of nurses include a lack of interest in the profession, high turnover rates, inadequate recruitment of young and efficient nursing staff, abandonment of service, low nursing salaries, insufficient managerial support, and migration of nurses [26]. The nursing director at SBMU provided information on the nurse-to-patient ratio, which is approximately 0.7, excluding certain departments such as operating rooms, labor, emergency, and dialysis where patients are hospitalized for less than 24 h. During the time of this study, nursing care is delivered through the case method in intensive care units and dialysis wards and the functional method in other wards.

OCBs are not included in undergraduate and postgraduate nursing curricula in Iran. Furthermore, Research findings indicate that Iranian nurse managers frequently use transformational leadership styles in nursing [27–29]. Transformational leadership involves leaders promoting change in line with their values and interests to help followers achieve expected goals. This type of leader is characterized by four traits: idealized influence, inspirational motivation, intellectual stimulation, and individual consideration [30]. Accordingly, Iranian nursing managers encourage positive relationships, cooperation, and support among nurses that may influence of their OCBs.

### Participants

The study participants were nurses working in different wards in the hospitals. A total of 20 eligible nurses were recruited using purposive sampling. The inclusion criteria were to have at least one year of work experience and to have a bachelor’s degree in nursing. In order to achieve maximum variation, male and female nurses of different ages and levels (i.e. nurses and nurse managers), with different work experiences, with bachelor and master degrees and employed in different departments were included.

### Data collection

To collect the data, semi-structured, in-depth interviews were conducted with the participants. Upon obtaining written informed consent, interviews with open-ended questions in line with the study objectives were practiced. The interview venue and time were further chosen as the participants liked. The interviews were managed in a quiet room inside the hospital departments, commonly in the nursing supervisor’s room. In total, 20 semi-structured interviews were completed, each one lasting 30–60 min. The interviews started with an open-ended question regarding OCB and nurses’ experiences, and then guided to reach the main objectives of the study according to their responses (Supl. 1). Exploratory questions were also raised to gain a deeper understanding. The interviews continued until information saturation was reached.

The participants’ responses to each interview question were additionally explored. By restating the main points or summarizing the responses, there was much attempt to confirm the completeness and accuracy of the data and increase the credibility of the results. The participants were ultimately asked to express any other experiences or additional information, and the interviews were recorded by a voice recorder.

### Data analysis

The data analysis was made using qualitative content analysis, as the most common method exploited in the studies aimed at describing the characteristics of a text and the related phenomena [31]. It could provide an integrated view of the text and thus help understand OCB as a social phenomenon in a subjective but scientific manner.

The initial stage of the process involved transcribing the interviews. Following this, the manuscripts were read several times to gain a comprehensive understanding of the text and achieve immersion. In the third stage, meaning units were determined. Each meaning unit was then assigned a code in the fourth step to organize the data in a clear and understandable way. Accordingly, a total number of 650 codes emerged. The codes were then categorized into sub-categories based on their similarities and differences. The final categories were extracted by classifying the sub-categories. The findings were then described and interpreted [32].

### Rigor

In this study, the four criteria of credibility, dependability, transferability, confirmability, as well as authenticity were recruited to find the scientific accuracy and validity of the data. For this purpose, the relationship between the study objectives along with the interview questions and participants’ responses were checked and confirmed by the research team. The researchers further tried to increase the rigor of the data by immersing in them for a long time and commencing the interviews based on the required data. The data coding and analysis, using simultaneous peer review by two researchers and checking the interview transcripts and initial codes by three participants (viz., member checking), were also among other measures to enhance data rigor. Providing a detailed report of the research procedure, including the demographic characteristics of the participants, the sampling type, the data collection method, the study location, and the representation of the results together with the participants’ quotations were also other measures taken to add to the rigor of the study [33, 34].

## Results

### Participants’ demographic characteristics

The study involved 20 participants, including 4 head nurses and 16 nurses, aged between 23 and 48 years old, with work experience ranging from 1 to 29 years. Nine participants held a bachelor’s degree and 8 held a master’s degree (Table 1).

**Table 1:** Characteristics of the Participants

Participant no.	Age	Gender	Position	Level of Education	Experience (years)	Ward
1	25	Female	Nurse	BSc	1	Medical
2	27	Female	Nurse	MSc	5	NICU
3	26	Male	Nurse	MSc	4	ICU
4	24	Male	Nurse	MSc	1	Pediatric
5	25	Female	Nurse	BSc	1	Medical
6	35	Female	Nurse	BSc	12	Pediatric
7	26	Male	Nurse	BSc	3	Emergency
8	25	Male	Nurse	BSc	1	Surgical
9	25	Male	Nurse	BSc	4	Medical
10	35	Female	Nurse	MSc	10	NICU
11	40	Female	Head nurse	MSc	16	ICU
12	45	Female	Nurse	BSc	28	Surgical
13	50	Female	Nurse	BSc	29	ICU
14	35	Female	Nurse	MSc	10	Medical
15	36	Female	Nurse	BSc	13	Surgical
16	40	Female	Head nurse	MSc	17	CCU
17	43	Female	Head nurse	BSc	25	CCU
18	36	Female	Head nurse	BSc	14	Medical
19	48	Female	Nurse	MSc	24	ICU
20	37	Female	Nurse	BSc	14	Angiography

### OCB categories and subcategories

The study yielded nine subcategories and three main categories: ‘helping behavior’, ‘extra-role behavior’, and ‘contribution to professional growth and development’. These categories will be explained in detail below (Table 2).

**Table 2:** Categories and subcategories of organizational citizenship behaviors of nurses

Categories	Subcategories	Codes
**Helping behavior**	Helping colleagues at work	Helping new-to-the-job colleagues; Assisting colleagues burdened with heavy workload; Lending a hand to tired colleagues; Aiding sick colleagues Answering colleagues’ questions during non-shift hours; Not taking a rest to help other colleagues Prioritizing colleagues’ tasks over their own
	Helping colleagues outside of work	Providing financial support to colleagues in need; Helping colleagues solve their familial problems
	Boosting morale	Making colleagues feel energized; Conveying a sense of value to colleagues and oneself
	Creating a culture of support and appreciation	Performing unrelated duties as needed; Cooperating in training students; Accepting more tasks once other colleagues are in short supply; Contributing to charitable organizations to purchase equipment
**Extra-role behavior**	Cooperation in advancing tasks	Performing unrelated duties as needed; Cooperating in training students; Accepting more tasks once other colleagues are in short supply; Contributing to charitable organizations to purchase equipment
	Creativity and efforts to promote services	Showing creativity in supplying deficiencies; Paving grounds for family-centered care conditions; Offering suggestions for improving physical environments; Maintaining equipment in departments
**Contribution to professional growth and development**	Individual professional development	Participating in educational workshops; Learning new skills
	Support for colleagues’ professional development	Helping to improve skills in colleagues; Creating educational groups among colleagues

### Helping behavior

Helping behavior refers to actions intended to assist colleagues either at work or outside of work, creating a positive atmosphere and appreciation for the help of colleagues. This finding consists of four subcategories: helping colleagues at work, helping colleagues outside of work, boosting morale, and creating a culture of support and appreciation.

### Helping colleagues at work

The study participants acknowledged that they had assisted new, sick, or fatigued colleagues, or even those with heavy workloads, to assume their own responsibilities. Some also stated that they had attempted to answer their colleagues’ questions about healthcare services by phone, even when they were not on duty. Participant No. 12, a nurse in the department of surgery with 25 years of work experience, stated that:

*I talk to my new colleagues all the time, I teach them a lot, I tell them not to feel intimidated and I help them as much as I can. I even try to reassure them that all nurses are the same. I help them to get to know the department better. I even introduce them to the department, show them where the necessary tools and equipment are and teach them how to use them.*

Participant No. 4, a nurse in the pediatric department, shared an experience of supporting a colleague during their illness:

*During a night shift, a colleague was finishing her evening shift and about to start the night shift. She was feeling unwell and tired, and her patients required more attention than mine. I stopped to help her feel less worried and even encouraged her to take a rest.*

### Helping colleagues outside of work

The participants identified helping colleagues with financial and familial problems as a key aspect of ‘helping behavior’. Participant No. 11, who heads an intensive care unit (ICU), discussed assisting colleagues with financial burdens as follows:

*In this department, we function like a family. We are familiar with these situations. If someone encounters a financial problem, we make every effort to assist in resolving it.*

### Boosting morale

Another subcategory of ‘helping behavior’ is boosting morale. According to the study participants, they aimed to share a positive mood with each other and create a supportive atmosphere by helping and energizing their colleagues. For instance, a nurse working in an internal medicine department stated:

“*In this department, I pay close attention to my colleagues and always offer a helping hand. I provide positive energy and strive to make them feel better. When my colleagues see that I am helping them, they reciprocate. Usually, this increases energy and boosts confidence.*” (Participant No. 5).

### Creating a culture of support and appreciation

The final subcategory of ‘helping behavior’ is creating a culture of support and appreciation in the workplace. According to the study participants, they found it challenging to cultivate a sense of gratitude and expand help through their own behavior. Participant No. 17, a supervisor in a coronary care unit (CCU), expressed this sentiment with the following quote:

*I aim to express gratitude to my colleagues, even for the small things they do or the efforts they make to advance the department’s work. This lets them know that their efforts are respected.*

### Extra-role behavior

“Extra-role behavior” refers to a range of behaviors and actions that go beyond the formal job description, including collaboration to advance tasks, creativity and efforts to promote services, and multifaceted patient/family support, as described below.

### Cooperation in advancing tasks

Participants in the study stated that they had worked much more collaboratively to progress tasks within the departments, taking on some responsibilities outside of the department, working as a team to teach students in clinical settings, taking on more tasks when other colleagues were absent, and contributing to charitable organizations to purchase some equipment. For example, participant No. 20, who works in the angiography department, verbalized that:

*Sometimes, when my colleagues leave their shifts for any reason, I collaborate as much as possible and take up the shifts to ensure that the department does not face any problems.*

### Creativity and efforts to promote services

One of the subcategories of ‘extra-role behavior’ was creativity and efforts to promote services. Some examples were showing creativity in overcoming shortages, providing health services, paving the way for family-centered care, and making suggestions for improving the physical environment and equipment maintenance. Some quotes related to dealing with shortages and thinking creatively in providing health services were as follows:

“*I remember a time when we needed to use nasal continuous positive airway pressure (nCPAP) for infants. However, the hat we had was worn out. As I knew how to sew, I made a new hat to fit the babies. So, I made some. There were also times when we needed to position the infants, but there was a shortage of nests, and the hospital could not provide them.*” (Participant No. 10, neonatal ICU nurse).

“*During the COVID-19 pandemic, we provided needleless injection vials to patients with breathing problems before administering injections. If the patients tolerated it well, their condition would improve, and we would proceed with the injection. However, this procedure had one drawback: patients became thirsty quickly and required frequent water breaks. To address this issue, we innovated by creating a small hole in the vial to allow for easy drinking water access. We attached a headset to the patient’s bed and placed it near their mouth so they could drink water whenever they felt thirsty. The patients appreciated this creative solution as they were previously frustrated with having to call for water. They welcomed this time-saving practice and felt better after using it.*” (Participant No. 13, ICU nurse).

### Contribution to professional growth and development

‘Contributing to professional growth and development’, as the third category of OCB, represented those instances where nurses engaged in organisational change through personal growth and development. There were two subcategories, named individual professional development and supporting the professional development of colleagues, as shown below.

#### Individual professional development

The study participants mentioned that they had attended educational workshops to improve their professional conditions and learn new skills. In this regard, Participant No. 9 said that:

*When I joined this department as a new member, I quickly realized the importance of learning about our patients, their diseases, and how to operate medical devices to provide quality healthcare services. To achieve this, I visited hospitals across the province and attended workshops to expand my knowledge. I am eager to continue learning and sharing my knowledge with colleagues.*

### Support for colleagues’ professional development

The participants emphasised the importance of their own professional growth and development, and believed in providing good opportunities for their colleagues. Participant No. 14, who has 10 years of work experience and a master’s degree, stated that:

*Many colleagues struggle with searching scientific databases. I taught them how to search for and read articles.*

## Discussion

The study revealed the experiences of Iranian nurses regarding OCB under three categories: ‘helping behavior’, ‘extra-role behavior’, and ‘contribution to professional growth and development’.

The first category of OCB, “helping behavior”, was demonstrated via helping colleagues at work, helping colleagues outside of work, boosting morale, and creating a culture of support and appreciation in their behavior. Two subcategories of helping colleagues at work and helping colleagues outside of work in this study were in parallel with altruism, introduced by Organ [8]. Moreover, two other subcategories, boosting morale and creating a culture of support and appreciation in the present study were reminiscent of courtesy [8]. There were also some similarities between the study findings and one of the OCB dimensions addressed by Podsakoff et al. [10], viz. helping behavior, which included helping others or preventing work-related problems voluntarily. The reviews further showed that most studies on nurses’ experiences of OCB in Iran and other countries had been conducted descriptively and cross-sectional. For example, a survey on the nurses of 15 teaching hospitals in Tehran, Iran, had indicated that the majority had shown OCB, particularly altruism, at a desirable level [16]. In another study in an Indonesian hospital, the nurses’ OCB had been high with reference to the model developed by Organ [35]. Considering that recurrent altruism could boost individual performance and reinforce teamwork, there were thus far more opportunities to improve employees’ knowledge [36]. In light of this, bolstering these behaviors by managers could help improve nursing care quality and patient outcomes.

The second category under OCB identified in this study was nurses’ “extra-role behavior” through cooperation in advancing tasks, creativity and efforts to promote services, as well as multifaceted patient/family support. By spending much more time and effort beyond their formal job descriptions, these nurses had accordingly tried to elevate organizational performance and deliver optimal healthcare services to patients. This was the same as conscientiousness, one of the OCB dimensions presented by Organ [8]. From his standpoint, conscientiousness meant displaying voluntary behavior by employees that might overdo the minimum requirements of their organizational roles [8]. Podsakoff et al. [10] had further mentioned individual initiative as OCB, which could be in the vein of “extra-role behavior”. In his opinion, individual initiative represented engagement in work-related behaviors far beyond the lowest required or expected levels on a voluntary basis. Such behaviors were creative, voluntary acts exhibited to improve organizational performance accompanied by enthusiasm and extra efforts to fulfill some tasks, volunteering for additional responsibilities, and encouraging others in an organization to do the same. All these behaviors had the impression that employees were “above and beyond” their duties [10]. Conscientiousness could refer to a set of voluntary behaviors demonstrated by nurses, as reported in other studies. In a survey in Turkey, the nurses working in a private hospital had thus displayed the highest levels of OCB in terms of conscientiousness [37]. Besides, Altuntas and Baykal [38] had found that nurses had shown conscientiousness more than other forms of OCB. In another study in Indonesia, the nurses working in Labuang Baji Hospital had been reported with a high level of conscientiousness [35]. Given that nursing was a stressful profession [39] and conscientiousness could be the most effective predictor of problem-solving in response to stressful factors [40], this type of OCB could help better manage the quality of nursing care.

The third category in this study was “contribution to professional growth and development”. The study results disclosed that the nurses had thus taken some steps to improve organizational performance through individual professional development and support for that in their colleagues. This could be comparable to one of the OCB dimensions, called civic virtue, described by Organ (1988) and self-development as one of the seven dimensions of OCB introduced by Podsakoff et al. [10], viz., a voluntary behavior performed by employees to improve ones’ knowledge, skills, and abilities. Although professional development obtained in this study was one of the dimensions of OCB and similar to those raised by other researchers, this voluntary behavior could facilitate the transition of roles in nursing. As reported in a cross-sectional study, self-development was a driving force for transferring from basic to professional nursing roles [41]. As well, Liu et al. [42] had found in their review study that personal growth was one of the positive emotional experiences and professional benefits in acute and critical care nurses. In another qualitative study, the results had shown that community health nurses could consider self-development as a strategy to build ones’ resilience [43]. According to the findings of this study and the previous ones, it was concluded that professional development as a dimension of OCB could result in other positive outcomes in nurses.

## Conclusion

This study was practical from two perspectives. First, there were limited studies about nurses’ experiences of OCB, as the key human resources in healthcare systems. This qualitative study was thus able to provide new insights about such a voluntary behavior. The second reason was the main concept addressed in this study, that is, OCB, as a relatively new concept affecting the quality of healthcare services, whose investigation could reveal new dimensions.

The study results established that the Iranian nurses had behaviors similar to what had been introduced as OCB in the related literature, including “helping behavior”, “extra-role behavior”, and “contribution to professional growth and development”. In this regard, nursing managers are suggested to dedicate much attention to OCB while employing and evaluating nurses. On the other hand, educational planners and nursing instructors should include OCB in nursing curricula and continuous training programs.

Among the major limitations in this study was no familiarity with the concept of OCB among the nurses during the interviews. For this purpose, the interviewer used some words resembling this concept in the interview questions. This study was also completed in Iran, but could be generalized to similar healthcare systems and cultural contexts. However, more studies should be conducted on nurses’ experiences of OCB through other qualitative methods, such as phenomenology, grounded theory, and ethnography to shed light on more dimensions in this field.

### Electronic supplementary material

Below is the link to the electronic supplementary material.

Supplementary Material 1

## Data Availability

The data will be made available from the corresponding author upon reasonable request.

## Abbreviations

OCB: Organizational citizenship behavior
SBMU: Shahid Beheshti University of Medical Sciences
