# Supplementary material for: Nurses’ experiences of the attributes of the organizational citizenship behavior: a qualitative content analysis
Source: BMC Health Serv Res. 2024 Apr 26;24:538. doi: 10.1186/s12913-024-10939-8 (PMC11055363; doi:10.1186/s12913-024-10939-8)
Supplement: Supplementary file 1 — Supplementary Material 1 [file 12913_2024_10939_MOESM1_ESM.docx]

The guiding questions in the interviews:

- What behaviors do you practice beyond your job description according to your experiences to move things forward better?
- How do you and your colleagues perform these behaviors in the work environment?
- What things help improve such behaviors?
- Is there anything else you would like to add?
- Is there another question I should have asked?
